# A multidisciplinary approach to the clinical management of Prader–Willi syndrome

**DOI:** 10.1002/mgg3.514

**Published:** 2019-01-29

**Authors:** Jessica Duis, Pieter J. van Wattum, Ann Scheimann, Parisa Salehi, Elly Brokamp, Laura Fairbrother, Anna Childers, Althea Robinson Shelton, Nathan C. Bingham, Ashley H. Shoemaker, Jennifer L. Miller

**Affiliations:** ^1^ Division of Medical Genetics and Genomic Medicine, Department of Pediatrics Vanderbilt University School of Medicine Nashville Tennessee; ^2^ Department of Psychiatry, Child Study Center Yale School of Medicine New Haven Connecticut; ^3^ Clifford Beers Clinic New Haven Connecticut; ^4^ Pediatric Gastroenterology Johns Hopkins Children's Center Baltimore Maryland; ^5^ Division of Endocrinology and Diabetes, Seattle Children’s University of Washington Seattle Washington; ^6^ Neuro‐Sleep Division, Department of Neurology Vanderbilt University School of Medicine Nashville Tennessee; ^7^ Division of Pediatric Endocrinology, Department of Pediatrics Vanderbilt University School of Medicine Nashville Tennessee; ^8^ Pediatric Endocrinology University of Florida Gainesville Florida

**Keywords:** genomic imprinting, interdisciplinary communication, outcome and process assessment (health care), Prader–Willi syndrome, telemedicine, translational medical research

## Abstract

**Background:**

Prader–Willi syndrome (PWS) is a complex neuroendocrine disorder affecting approximately 1/15,000–1/30,000 people. Unmet medical needs of individuals with PWS make it a rare disease that models the importance of multidisciplinary approaches to care with collaboration between academic centers, medical homes, industry, and parent organizations. Multidisciplinary clinics support comprehensive, patient‐centered care for individuals with complex genetic disorders and their families. Value comes from improved communication and focuses on quality family‐centered care.

**Methods:**

Interviews with medical professionals, scientists, managed care experts, parents, and individuals with PWS were conducted from July 1 to December 1, 2016. Review of the literature was used to provide support.

**Results:**

Data are presented based on consensus from these interviews by specialty focusing on unique aspects of care, research, and management. We have also defined the Center of Excellence beyond the multidisciplinary clinic.

**Conclusion:**

Establishment of clinics motivates collaboration to provide evidence‐based new standards of care, increases the knowledge base including through randomized controlled trials, and offers an additional resource for the community. They have a role in global telemedicine, including to rural areas with few resources, and create opportunities for clinical work to inform basic and translational research. As a care team, we are currently charged with understanding the molecular basis of PWS beyond the known genetic cause; developing appropriate clinical outcome measures and biomarkers; bringing new therapies to change the natural history of disease; improving daily patient struggles, access to care, and caregiver burden; and decreasing healthcare load. Based on experience to date with a PWS multidisciplinary clinic, we propose a design for this approach and emphasize the development of “Centers of Excellence.” We highlight the dearth of evidence for management approaches creating huge gaps in care practices as a means to illustrate the importance of the collaborative environment and translational approaches.

## 1 INTRODUCTION

Prader–Willi syndrome (PWS) is a multisystem complex disorder present in 1/15,000–1/30,000 individuals that is caused by the loss of paternally expressed genes on chromosome 15q11.2‐13. Persons present with significant hypotonia and poor feeding until approximately 9 months old and progress through nutritional phases, including a period of improved feeding and appetite with normal growth, weight gain without increased appetite, increased appetite and calorie intake with satiety still possible, hyperphagia, and sometimes satiety in adulthood (Miller, Lynn, Driscoll, et al., 2011).

Endocrinological abnormalities include hypogonadism, short stature due to growth hormone deficiency, central hypothyroidism, adrenal insufficiency, premature adrenarche, and osteopenia. Hypoglycemia may be underappreciated, especially in infants. Sleep disorders such as apnea and excessive daytime sleepiness are common. Developmental delays, including mild intellectual disabilities and speech and articulation defects, are also prevalent. Signature behaviors include tantrums, poor transitioning, obsessive–compulsive tendencies, autistic‐like features, and skin picking. Comorbid psychiatric diagnoses include major depressive disorder with or without psychotic features, bipolar disorder with psychotic features (UPD specific), autism (Dykens, Roof, Hunt‐Hawkins, Dankner, et al., 2017), and psychotic illness (Soni et al., 2007). The physical examination usually reveals typical facies such as almond‐shaped palpebral fissures and a narrow bifrontal diameter, hypotonia, small hands and feet, strabismus, scoliosis, and thick saliva.

The American Academy of Pediatrics published clinical guidelines in 2011 (McCandless & Committee on Genetics, 2011). Global developmental delay and hypotonia are sensitive for diagnosis (Gunay‐Aygun, Schwartz, Heeger, O'Riordan, & Cassidy, 2001). Additional features that should prompt testing may differ depending on the age of the patient. In infancy, these include lack of interest in oral feeding, assisted feeding with a gastrostomy or nasogastric tube, and hypoplastic genitalia. In older children, additional evaluative features include an inability to achieve satiety, hypogonadotropic hypogonadism, and characteristic behaviors such as skin picking. Short stature relative to genetic background and degree of obesity may also be an indication for testing. Methylation analysis detects 99% of cases. Younger age at diagnosis enables interventions and has changed the natural history of the disease. Additional recognized features include progression through the nutritional phases, highlighting the importance of a low threshold for diagnosis, particularly in children with behavioral concerns.

There is little evidence regarding the role of multidisciplinary clinics in the management of rare diseases, but success is evident in changes in practice for certain conditions in particular in cystic fibrosis and sickle cell disease. With respect to PWS, models of these approaches exist in France and the Netherlands, and this has facilitated research approaches to understand the natural history of disease (Molinas et al., 2008). Quantifying the value of a multidisciplinary clinic should not be based on revenue alone. It is also important to recognize the importance of enhanced quality of care provided and the ability to participate in translational research filling the gaps in knowledge. Data available regarding integrated healthcare models suggest improved treatment outcomes and reduced healthcare costs (Unutzer et al., 2002, 2008 ). To date, these models include primary care physicians and behavioral health specialists working together often on a referral basis. Where these models fall, short is real‐time coordination of care in which specialists see the patient during the same clinic visit.

We propose a multidisciplinary approach to deliver comprehensive care in a setting where specialists are collocated, communicate closely, and deliver patient‐ and family‐centered care. Much of the literature on PWS focuses on management of symptoms and proposes this approach (Angulo, Butler, & Cataletto, 2015; Cassidy & Driscoll, 2009; Deal et al., 2013; Goldstone, Holland, Hauffa, Hokken‐Koelega, & Tauber, 2008). However, recent data regarding morbidity and mortality for this disorder highlight significant knowledge gaps. Based on our experience with a multidisciplinary clinic and review of the literature, we aim to expand upon proposed models (Grosse et al., 2009; Kerem, Conway, Elborn, Heijerman, & Consensus, 2005), including add up‐to‐date approaches, and broaden the purview to develop “Centers of Excellence”.

We propose that designated Centers of Excellence would include the multidisciplinary clinic, collocated collaborative bench researchers, coordination of research from the bench to the bedside, clinical research programs to establish evidence‐based standards of care through rigorous clinical research, a coordinating center to provide advice to community providers, families, and industry interested in pursuing PWS research programs. It should include using updated technologies such as telemedicine to improve access to care, using a common data model through a global registry, facilitating new research ideas and translational advances, and bringing new treatments or providing concrete evidence for care practices to patients through randomized clinical trials. We propose bringing together medical providers, researchers, pharma, and families to help the field progress through a network of Centers of Excellence across the globe. Clinical directors of Centers of Excellence should meet regularly to ensure standards of care between providers. We propose recognition and support from a governing organization that would include members from all with an investment in the care of individuals with PWS including parents, pharma, scientists, clinicians, and ancillary medical staff among others. A similar guideline to that established by the Huntington Disease Society is rigorous and ideal for providing the best‐standardized care (https://hdsa.org/).

A table summarizing medical care is provided (Table 1) and constitutes an expansion of AAP guidelines (McCandless & Committee on Genetics, 2011).

**Table 1:** Medical checklist for providers managing PWS

Age	Medical Eval	Anticipatory guidance	Medical referrals	Labs	Diagnostic	Medication/supplement considerations
Newborn infants	Feeding Growth & Dev't Tone Gonadal dev't^a^	<20 min per feeding Diet has appropriate macro‐ and micronutrients development Early intervention services Sufficient environmental stimulation Support groups	Genetics Endocrine Ophthalmology^b^ Pulmonary Nutrition OT, PT, ST Neurology, Urology prn	IGF1, IGFBP3 Thyroid studies (free T4 and TSH)^d^ 25‐OH vitamin D	PSG (before starting GH and 8–12 weeks after starting) Echocardiogram if murmur (4% CHD) Hip US (DDH) Feeding eval	Growth hormone* Multivitamin Trial the supplements^c^
1 month–1 year	Growth & dev't Gonadal dev't Vision (strabismus) Feeding	Early interventional services Cognitive dev't Routines Limit‐setting Seizures (5%–10%) Support groups	Endocrine Genetics Ophthalmology^b^ Pulmonary Nutrition ST, OT, PT Audiology Neurology, urology prn	IGF1^d^, IGFBP3^d^ Thyroid studies^d^ CBC B12 level prn Adrenal eval 25‐OH vitamin D iron studies	PSG as above Swallow study EEG prn Scoliosis screening X‐ray (when can sit up with core) X‐ray hip (DDH)	Growth hormone* HCG in males with cryptorchidism (3–18 months old); consider orchiopexy Multivitamin Trial the supplements^c^
1–5 years old	Growth & dev't Vision Hearing Feeding Scoliosis Sleep Behavior Gastroenterology/nutrition	Dental Care (increased risk of caries) Early intervention Behavior Socialization Access to food and environmental modifications for the family Constipation Slowed metabolism Routine Limit‐setting Support groups DDA	Endocrine Genetics Ophthalmology^b^ Pulmonary Nutrition ST, OT, PT hippotherapy aquatic, and others Behavioral specialist Dentist^e^ Neurology prn	IGF1^d^, IGFBP3^d^ Thyroid studies^d^ Calcium Electrolytes CBC B12 prn Adrenal eval Lipid panel 25‐OH vit D iron studies	PSG prn Swallow evaluation prn EEG prn Scoliosis screening X‐ray NP eval	Growth hormone* HCG in males with cryptorchidism (3–18 months old), consider orchiopexy Trial of supplements^c^
5–13 years old^j^	Growth & dev't Pubertal dev't (premature adrenarche, precocious puberty) Vision Skin examination (picking) Hearing Feeding Scoliosis Sleep Behavior Gastroenterology/nutrition	Access to food as noted Socialization Routine Limit‐setting Constipation Weight management Behavior OSA Daytime sleepiness High pain tolerance Gastric necrosis Support groups DDA	Endocrine Genetics Ophthalmology^b^ Pulmonary Nutrition ST, PT, OT hippotherapy aquatic, and others Behavioral specialist Neurology prn Dentist^e^	Thyroid studies,^d^ IGFI,^d^ IGFBP3^d^ Hemoglobin A1C Fasting insulin and glucose Calcium Electrolytes CBC B12 prn Adrenal eval Lipid panel Pubertal eval as indicated	PSG prn Feeding eval MSLT prn PFTs EEG prn Scoliosis screening X‐ray NP eval	Growth hormone* modafinil^g^ stimulant^f^ *N*‐acetylcysteine SSRIs^h^ Antipsychotics Metformin supplements^c^
13–21 years old	Cardio‐vascular examination (at risk for heart failure) if obese Skin examination (Picking) Pubertal dev't Scoliosis Feeding Exercise Psychosis Behavior Gastroenterology/nutrition	Routine Skin care Behaviors (e.g., psychosis) School placement Gastic necrosis Gynecologic care Constipation Weight management Access to food as noted Sexual/behavioral health Socialization Guardianship Transition of care Support groups DDA	Endocrine Genetics Ophthalmology^b^ Pulmonary Nutrition ST, PT, OT, hippotherapy aquatic, and others Behavioral specialist Psychiatry prn Neurology prn Dentist^e^	Thyroid studies^d^ IGF1,^d^ IGFBP3^d^ Fasting insulin and glucose Hemoglobin A1C Oral glucose tolerance test Calcium CBC Electrolytes B12 prn Lipid panel Adrenal eval Pubertal eval^j^	PSG prn Feeding eval DEXA MSLT prn EEG prn Pelvic US assess uterine lining Scoliosis screening X‐ray NP eval	Growth hormone modafinil^g^ stimulant^f^ *N*‐acetylcysteine SSRIs^h^ antipsychotics HRT Metformin GLP−1 receptor agonists^i^ supplements^c^
Adult	Cardio‐vascular examination (at risk for heart failure) Skin exam Weight Psychosis Sleep Behavior Gastroenterology/Nutrition	Routine Skin care Behavior (e.g., psychosis) Risk intestinal necrosis Daytime sleepiness Gynecologic care Socialization Constipation Weight management Guardianship Transition of care Support groups DDA	Endocrine Genetics Ophthalmology^b^ Pulmonary Nutrition ST, PT, OT, hippotherapy aquatic, and others Behavioral specialist Psychiatry prn Neurology prn Dentist^e^	Thyroid studies^d^ IGF1 (at least yearly) Hemoglobin A1C Fasting insulin and glucose Oral glucose tolerance test Calcium Electrolytes CBC B12 prn Lipid Panel Adrenal eval Pubertal eval^j^	PSG prn Swallow study prn DEXA MSLT prn PFTs EEG prn Pelvic US as above X‐ray to monitor scoliosis	Growth hormone HRT modafinil^g^ stimulant^f^ antipsychotics *N*‐acetylcysteine SSRIs^h^ Metformin GLP−1 receptor agonists^i^ supplements^c^

Note

AI: adrenal insufficiency; CBC: complete blood count; CHD: congenital heart disease; CoQ10: coenzyme Q10; DDA: developmental disabilities administration; DDH: developmental dysplasia of the hip; dev't: development; DEXA: dual‐energy X‐ray absorptiometry; DHA: docosahexaenoic acid; DHEA‐S: dehydroepiandrosterone sulfate; EEG: electroencephalogram; Eval: evaluation; FSH: follicle stimulation hormone; GGT: glucose tolerance test; GH: growth hormone; GLP‐1: glucagon‐like peptide‐1; HCG: human chorionic gonadotropin; HRT: hormone replacement therapy; IGF1: insulin‐like growth factor 1, somatomedin C; IGFBP3: insulin‐like growth factor‐binding protein 3; LH: luteinizing hormone; MCT: medium‐chain triglycerides; MSLT: multiple sleep latency test; NP: neuropsychological eval; OCD: obsessive–compulsive disease; OSA: obstructive sleep apnea; OT: occupational therapy; PFT: pulmonary function tests; prn: as needed; PSG: polysomnography; PT: physical therapy; PWS: Prader–Willi syndrome; SSRI: selective serotonin reuptake inhibitor; ST: speech therapy; T4: thyroxine; TSH: thyroid stimulation hormone; US: ultrasound.

* FDA approved treatment.

a Assess if testes are descended or whether an inguinal hernia is present.

b As needed (at risk for strabismus, cataracts and/or nystagmus).

c Trial of supplements (one at a time) such as CoQ10, Carnitine, MCT oil, DHA, B12 particularly if low levels are detected.

d At least once yearly (or if symptomatic for thyroid disease).

e More frequent cleaning every 3–4 months.

f If child has attention deficit symptoms and/or impulsivity.

g Based on behavior and sleepiness during the day.

h For behavioral concerns, depression, anxiety particularly if severe.

i For treatment for insulin resistance on case‐by‐case basis.

j Consider on case‐by‐case basis: Testosterone (total, male), Prolactin, DHEA‐S (adrenarche), FSH/LH (ultrasensitive for precocious puberty), Estradiol (female), anti‐Mullerian hormone, Inhibin B

## 2 MATERIALS AND METHODS

Interviews with expert medical professionals, scientists, managed care experts, parents, and individuals with PWS were conducted from July 1, 2016 to December 1, 2016. Either from the participation of medical professionals or families, 10 multidisciplinary clinics across the United States were represented in addition to many specialty providers across the country not working in a specifically established clinic. The responses of these 60‐ to 120‐min open‐ended interviews were compiled to formulate a care model for PWS. We assessed the unmet medical needs of families by asking specific open‐ended interview questions to assess whether specific standards were established in the care of their loved one with PWS. At the conclusion of the interview process, the collective expertise of the group was utilized to compile an approach to the management of PWS.

### 2.1 Ethical compliance

Personal interviews were conducted with colleagues in the field as stated and Institutional Review Board approval was not required. Institutional Review Board approval has been obtained for the collection of data through the Vanderbilt University Medical Center.

## 3 A MULTIDISCIPLINARY APPROACH

Multidisciplinary care improves upon fragmented care and serves as a medical home for patients. Services provided on a regular basis for coordinated and specialized care focus on disease‐specific surveillance, management of complications, and psychosocial needs of patients and families. Components of a team may include multiple medical providers and ancillary support, including case managers, administrative support for managed care coordination, early intervention services, nurses, physical, occupational and speech therapists, and social workers (Grosse et al., 2009; Kerem et al., 2005).

Advantages of such clinics are patient‐ and family‐centered coordination of care and communication to the medical home, a single appointment day leading to fewer missed days of work and school, opportunities for disease‐specific anticipatory care to prepare families for and prevent possible complications, and improved morbidity and mortality. In addition, open communication between providers optimizes care and may lead to a decreased cost of medical care—a clear concern for the medical profession and society at large.

Multidisciplinary clinics are not without drawbacks. Reimbursement may impede their emergence, particularly with fee‐for‐service‐based care, as health insurance restrictions may limit provider reimbursement in a single day. There is minimal reimbursement for coordination of care and individuals may need to travel great distances to these centers and therefore incur out‐of‐network costs. However, these costs may be offset by less total time spent in seeking health care through consolidated appointments. Involvement of managed care teams to negotiate directly with insurance companies regarding reimbursements associated with multidisciplinary care clinics is one proposed model to offset cost and financial burden on families subject to multiple copays. When worth is based on revenue, it devalues the importance of high quality and coordinated care by medical experts in the field, particularly when treating rare complex diseases.

Unfortunately, little data are published with regard to pertinent multisystemic rare diseases, perhaps due to the barriers noted. However, improved patient outcomes due to a multidisciplinary approach have been observed in pediatric chronic kidney disease, sickle cell disease, recalcitrant aerodigestive complaints, and diabetes management, among others (Ajarmeh, Er, Brin, Djurdjev, & Dionne, 2012; Asare et al., 2017; Chung et al., 2017; Rotsides et al., 2017). Compared to a control group, a group of pediatric chronic kidney disease patients in British Columbia, Canada, showed improved outcomes, particularly in anemia management, bone mineral metabolism, nutrition, and renal disease progression. Maternal morbidity was reduced for women with sickle cell disease living in under‐resourced areas. A multidisciplinary team reduced recalcitrant aerodigestive complaints in 73% of patients compared to being seen by a single specialist. Expanding the disciplines on a team improved treatment of problems related to medication adherence in diabetic patients. A multidisciplinary approach may also improve transition of care to adult services and adolescent transition to independence (Geerlings, Aldenkamp, Gottmer‐Welschen, van Staa, & de Louw, 2016).

## 4 MULTIDISCIPLINARY CLINIC WORKFLOW

**Figure 1:**
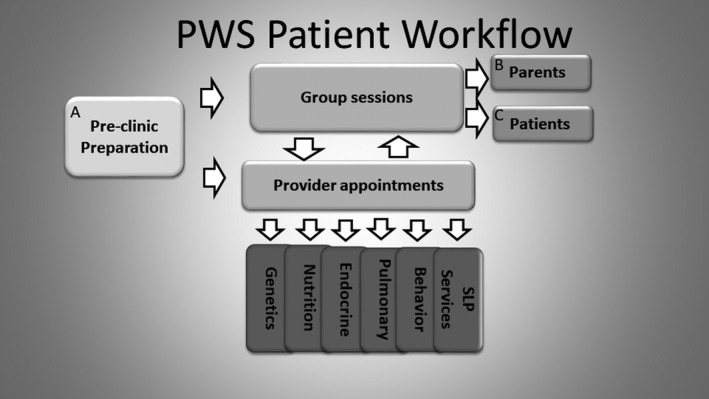
Example multidisciplinary clinic workflow. (A) Includes intake sheets and questionnaires linked to participation in a registry for the collection of worldwide data, synopsis prioritizing problem list created, and providers meet to plan personalized patient agenda with coordination of studies. (B) Topics are age‐based and family‐directed. (C) Includes group sessions with patient‐directed topics

One important aspect of a successful multidisciplinary clinic is the preparation for the clinic visit (Figure 1). Intake from families via surveys is key to prioritizing provider visits and patient problem lists. In addition to a history of present illness, prominent concerns from the family and patient, a 24‐hr diet recall, a hyperphagia questionnaire, sleep scales, and psychosocial, behavioral, and quality‐of‐life assessments should be included. If possible, coordinating with a collaborative global registry for uniform collection of data between centers is important, as it can lead to progress in defining disease natural history (www.pwsregistry.org).

Clinical opportunities should include group sessions for parents and patients. Topics of interest could be surveyed during the initial intake. For infants to school‐age children, one possibility for parental group sessions is discussion of typical versus PWS‐specific behaviors. Distractions and activities for patients through involvement of Child Life may be beneficial to allow parental participation in group sessions and individualized time with medical providers (Figure 1). Additional providers available may include an ophthalmologist and a neurologist, the latter particularly if there is concern for seizures. Orthopedics may be needed for consultation regarding hip dysplasia and scoliosis. Urological evaluation is needed for cryptorchidism and genetic counselors should be available for new diagnoses and family planning.

## 5 CENTERS OF EXCELLENCE

Multidisciplinary clinics facilitate growth of Centers of Excellence. We propose this term as an all‐encompassing initiative that includes the multidisciplinary clinic integrated with research as well as educational programs for families and medical and affiliated professionals. Specifically, such a Center's role is multifaceted and should encourage clinical work that informs translational research to elucidate natural history alongside pathophysiology and brings novel targeted therapies directly to patients through clinical trials. Its role is integral in developing clinical outcome measures and biomarkers of disease to study objective success of therapies. In particular, it is important in the formation of a network to conduct randomized control trials. It should provide local, national, and international resources to scientists, families, pharma, and medical professionals.

## 6 THE PWS CENTER DIRECTOR

A multidisciplinary team involves a director with expertise in PWS with a vision of leadership that includes high standards of care and a translational approach to elucidate the mechanism of each phenotypic aspect of disease. The tasks at hand are of dual importance and include providing care aimed at addressing the patient's needs and the family's concerns, and trying to bridge the gaps in medical knowledge and satisfying unmet patient needs by creating and implementing standardized measures and running clinical trials efficiently and safely. The director should have a thorough knowledge of the literature, issues in the community, including patient‐initiated nutritional and treatment trials, and availability of clinical trials. They should address potential questions from families, including those related to the psychosocial and emotional impact of diagnosis.

Important coordinating responsibilities to consider:

- Providing anticipatory guidance based on the current knowledge of the natural history of PWS
- Ensuring individuals with PWS have a disease card and medical alert booklet that includes comorbidities, diagnoses, treatments, reference center numbers, and therapeutic indications and contraindications; for PWS, a book is available through Prader–Willi Syndrome Association USA.
- Advocating for patients, including ensuring appropriate individualized education plans in school and early intervention services
- Responding to questions and concerns from families
- Facilitating communication between providers, including hosting regular team meetings to review patients’ health status
- Communicating with patients’ primary care physicians and other medical providers
- Serving as a resource in the management of symptoms, such as behavioral concerns
- Providing global access to comprehensive care through the utilization of telemedicine
- Coordinating and remaining current regarding research and clinical trials
- Auditing the team's performance and practices
- Participating in a national and international registry to collect and share data
- Biobanking of samples to encourage sharing and formation of global translational research programs
- Communicating with basic researchers regarding clinical experiences toward the development of comprehensive bench‐to‐bedside initiatives
- Initiating research with colleagues in the community to contribute to multicenter national and international studies
- Developing a local, national, and international education program for medical professionals and families
- Working with local and national parent organizations and support groups to unite families, plan events and conferences, raise funds, and promote awareness.
- Advocating for legislative changes and access to longterm care for individuals with PWS.

## 7 THE MULTIDISCIPLINARY TEAM

The PWS multidisciplinary team should include at a minimum a geneticist, endocrinologist, a nutritionist and/or gastroenterologist, a sleep and pulmonary medicine physician, a behavioral and/or neurodevelopmental specialist, and interventional services including speech/language therapy.

## 8 THE PWS GENETICIST/GENETIC COUNSELOR

The role of the geneticist is initially essential to provide diagnosis including of the molecular subtype. Counseling provides information based on subtype and explanation of recurrence risk. Individuals with deletion or uniparental disomy (UPD) have a low risk of recurrence (<1%) in most cases. However, if there is an unbalanced chromosomal rearrangement, the risk of recurrence of the deletion subtype is increased. Additionally, in cases of UPD, a parental unbalanced translocation or marker chromosome increases the risk of recurrence. Special studies are required to diagnose uniparental heterodisomy; however, uniparental isodisomy may be diagnosed on chromosomal microarray. One could consider a karyotype in the instance of a diagnosis of uniparental isodisomy such as on chromosomal microarray to rule out a Robertsonian translocation.

Deletions are detected by chromosomal microarray and can be divided into two subtypes. Type I deletions span breakpoint (BP)1 and the distal BP3 (chr15:22,876,632_28,557,186; hg19). Type II deletions are smaller and span BP2 and BP3 (chr15:23,758,390_28,557,186); hg19 reference genome. A balanced translocation in the father can increase risk of recurrence of a deletion (Driscoll, Miller, Schwartz, & Cassidy, 1993).

An imprinting defect could carry as high as a 50% recurrence risk in the case of a paternally inherited deletion in the imprinting center. An epimutation (nondeletion) in the imprinting center, which causes abnormal transcriptional repression of the paternally active genes, carries a low recurrence risk (<1%; Driscoll et al., 1993).

The geneticist may provide anticipatory guidance to families regarding common features of PWS. She/he may also be the first to recommend enrollment in early intervention therapies at the time of evaluation or once diagnosis is confirmed. Another important example of anticipatory guidance includes information regarding thick and sticky saliva that warrants more frequent routine dental cleaning every 3 months due to an increased risk of dental caries. The geneticist should be familiar with what is known regarding the natural history of disease and possible genotype–phenotype correlations to begin providing a framework for families to prepare as their children grow and develop.

## 9 THE PWS ENDOCRINOLOGIST

The associated hypothalamic dysfunction present in PWS causes multiple endocrine abnormalities requiring the involvement of a knowledgeable endocrinologist (Diene et al., 2010). Growth hormone deficiency is common (Eiholzer et al., 1998). The prescribing endocrinologist should be able to effectively discuss and communicate the benefits and risks of treatment with rGH. It is most effective when started early to ensure appropriate brain development before the age of 1. This benefit is thought to be due to the role of insulin‐like growth factor 1 (IGF‐1) on brain development and maturation (Dyer, Vahdatpour, Sanfeliu, & Tropea, 2016). In a multidisciplinary setting, endocrinologists should consult with their nutritional, pulmonary, and otolaryngology colleagues to prevent, monitor, and treat conditions such as marked undernutrition in early life and obesity, premature adenarche, type II diabetes, and obstructive sleep apnea, that would otherwise prevent treatment with rGH (Souza & Collett‐Solberg, 2011). Routine monitoring of IGF‐1 levels should be used to titrate the rGH dosage to optimize benefits and limit risk. Endocrine abnormalities may lead to behavior changes, requiring the consultation of a psychiatrist to treat comorbid conditions.

Hypogonadism is a consistent feature manifesting as genital hypoplasia early in life and delayed/incomplete puberty in adolescence and adulthood. Given the prevalence of cryptorchidism in male patients, human chorionic gonadotropin (HCG) should be considered as well as consultation with an experienced pediatric urologist for surgical correction which is often necessary. At an appropriate age, sex steroid therapy may be considered for the induction of puberty with dose augmentation done in such a way that it reflects normal pubertal cadence. Considerations include the potential risks of thromboembolism and worsening behaviors. In addition, bone density should be monitored starting around the age of pubertal development (age 14). Hormone replacement therapy benefits bone density (Eldar‐Geva, Hirsch, Pollak, Benarroch, & Gross‐Tsur, 2013). There are cases of pregnant PWS females reported, which confers a risk of Angelman syndrome in offspring in the case of the deletion subtype. Risk of pregnancy may correlate with inhibin B and these levels should be checked with other hormones (Table 1). It remains unknown if some males produce sperm, which is conceivable in the minority that produce adequate testosterone. There are no reports to our knowledge of males conceiving.

As central hypothyroidism may develop, thyroid function should be screened for the first several months of life and at least annually thereafter, with a low threshold to initiate treatment should hypothyroxemia develop (Vaiani et al., 2010). Unfortunately, the incidence of central adrenal insufficiency varies significantly depending on the methodology for screening, and therefore, there is less consensus regarding appropriate screening for this disorder (de Lind van Wijngaarden, 2008). There is some evidence that patients with PWS have normal baseline cortisol levels, while their ability to mount a stress response may be compromised (Farholt, Sode‐Carlsen, Christiansen, Ostergaard, & Hoybye, 2011). As such, providers should consider screening adrenal function with provocative testing and/or prescribing stress dose steroids for use during significant illness, or before surgical procedures if the situation does not permit for prior testing. Hypoglycemia particularly in infants may be underappreciated and require monitoring. Finally, type II diabetes is a concern in obese individuals with PWS.

Thirst and temperature regulation are disordered in PWS. Preparation for outdoor activities and during extreme temperatures requires preparation including appropriate clothing, resting when tired and additional intake of fluids especially during participation in sports. Body temperature does not increase during infection and therefore antibiotic therapy should not be delayed if suspicion for infection is present.

## 10 THE PWS NUTRITIONIST/GASTROENTEROLOGIST

Important lifelong considerations for individuals with PWS include hyperphagia, lack of food selectivity, high pain tolerance, and a propensity for acute gastric dilation and necrosis. However, in infancy, different issues, such as feeding and swallowing difficulties, predominate. Special feeding techniques are often used to manage infant feeding difficulties. A gastrostomy tube should be avoided if possible, as it may affect the integrity of the stomach, which can increase long‐term morbidity and mortality. Nissen fundoplication in particular requires consultation with a pediatric gastroenterologist due to the known long‐term issues concerning dysphagia and esophageal clearance that are likely exacerbated by this procedure.

Another important consideration is the increased threshold for vomiting (Alexander, Greenswag, & Nowak, 1987). Vomiting does not occur under typical circumstances, and episodes of vomiting are important to take as urgent matters that may require emergency care. Emetics may be ineffective and toxic if repeat doses are administered. This is of particular concern when nonfood, spoiled or dangerous items have been consumed.

Aspiration and choking concerns are likely underdiagnosed. Increased morbidity and mortality is related to choking due to risk of asphyxiation. Clinical suspicion should prompt swallowing evaluation. Individuals with PWS require monitoring during feeding and should be encouraged to chew and eat slowly. Consumption of a meal should require intake of a glass of water prior to serving food. Thirst disorders are common in PWS. Individuals prefer other beverages to water in general.

An experienced pediatric nutritionist should see each child at every visit, while a speech pathologist can assist with swallowing difficulties. A regular diet should consist of appropriate fat intake for the developmental stage of the child. The best diet for each child should be based on their weight for length, rate of weight gain, growth velocity, and energy level during the day. Intake in infants is often at the lower end of normal for age. Infants should consume solely breast milk or formula until approximately 6 months of age. At 6 months, purees may start slowly at a tablespoon and be increased over a few months. As in typically developing children, a new food should be introduced on a weekly basis. At approximately 10 months of age, formula may decrease to no more than 8 ounces 3–4 times per day. We recommend families begin to modify the diet for all family members to ensure appropriately balanced nutrition.

Close oversight of diet throughout life ensures adequate provision of macronutrients (especially protein) with attention to micronutrients. Vitamin and/or mineral supplementation to meet the daily recommended intake by age may be necessary to prevent deficiencies.

Constipation is common in PWS and requires patient‐tailored management due to underlying dysphagia and baseline fluid intake. We recommend integration of sufficient fiber as part of a healthy balanced diet.

Following complex diets and restricting food is a lifelong commitment that requires environmental modifications such as locks on pantries to prevent access to food once individuals enter into nutritional stage 3. Education of all caretakers, including school personnel, is very important. Standard school meals may result in marked weight gain. To date, it has been recommended individuals follow a strict schedule to prevent food insecurity; however, it is possible this promotes compulsion to eat.

## 11 THE PWS SLEEP SPECIALIST

Several publications offer some characterization of sleep, including altered sleep architecture and reduced REM latency consistent with narcolepsy (Lassi et al., 2016). Polysomnography studies are essential due to both central and obstructive sleep apnea (OSA; Priano et al., 2006). Growth hormone therapy was initially thought to worsen OSA due to adenotonsillar hypertrophy possibly leading to sudden death; however, retrospective reviews of sudden death incidents in individuals with PWS on and off growth hormone showed no difference in the rates of death. Sudden death occurred within the first 9 months of growth hormone (GH) treatment in those who had sudden death on GH (Nagai et al., 2005). Guidelines for growth hormone therapy require polysomnography before starting growth hormone and 8–10 weeks after initiating treatment. In our clinical experience, rapid onset of sleep and reduced REM sleep latency consistent with narcolepsy or cataplexy are often present. Multiple sleep latency testing should be considered in individuals with symptoms of excessive daytime sleepiness despite treatment of OSA (De Cock et al., 2011). However, MSLT is often complicated by residual untreated OSA that inhibits ability to interpret results. Sleep abnormalities often require management with oxygen or CPAP. Untreated moderate‐to‐severe OSA is a risk for flash pulmonary edema particularly in settings of hypoxemia or extreme negative intrathoracic pressure. Close collaboration with an endocrinologist, nutritionist, and possibly behavioral specialist is important in addressing obesity‐related OSA. Behavioral specialists may also be helpful in training individuals to comply with the use of CPAP.

Patients may have recurrent respiratory infections, a significant cause of morbidity and mortality (Einfeld et al., 2006). Respiratory failure is a leading cause of death and if possible monitoring with pulmonary function tests to identify those that may be at risk should be considered. Higher rates of thromboembolism than the general population have also been described, and therefore the risk and clinical symptoms should be discussed as part of the anticipatory guidance especially in those individuals who are obese and/or have lower extremity edema.

## 12 THE PWS NEURODEVELOPMENTAL AND BEHAVIORAL SPECIALIST

Many of the behaviors and compulsions require family‐based therapeutic approaches to help keep the family functioning as a unit. A neurodevelopmental specialist such as a developmental pediatrician or a behavioral psychologist could be involved on an individual basis to address social functioning, anxiety, compulsive behaviors and stubbornness, and address developmental concerns. In addition, there is a role for age‐delimited group sessions to better address parents’ questions about behavior as noted above. With less access, particularly to mental health providers familiar with PWS, telemedicine coordinated through a PWS multidisciplinary clinic may play a role in assisting with such treatment. Toddlers and their families would particularly benefit from group sessions to provide appropriate expectations. For example, while families may attribute tantrums to PWS, it may provide reassurance to know that this is normal toddler behavior.

A behavioral specialist is invaluable for managing behaviors at different stages and providing recommendations regarding Individual Education Plans (IEP), school accommodations, and autism screening. Appropriate school placement is invaluable to families to minimize outbursts and maximize educational potential. Special attention is needed to restrict access to food, remove visible food items including trash from the individual's environment. One‐on‐one supervision is often needed. Additionally, neuropsychological testing may be helpful prior to school entry. A diagnosis of autism can help with obtaining ABA therapy, which can be beneficial if ABA therapists are trained to provide appropriate reward and training to respond to environmental cues including the presence of food items.

The availability of a consulting psychiatrist is helpful when additional input for medication management is needed that is beyond the comfort of the clinical team.

## 13 THERAPIES

In addition to access to early interventional services, a team approach should include the participation of physical, occupational, and speech therapists. Occupational and speech therapists can address common problems in infancy, such as feeding difficulties that often require the use of special nipples, and sensory integration. In addition, hypotonia is present throughout life and resulting difficulties will need to be assessed as needs shift during the different stages of growth and development. In infancy and childhood, there is an increased risk of gross and fine motor delays. Regular assessments can ensure the child is equipped with the best tools to succeed in the school system. In adulthood, involvement of occupational therapy helps with transitioning to independence in areas such as self‐care and performing other activities of daily living. An example of a therapy that is beneficial to build core muscle strength, balance, and coordination is hippotherapy. Other concerns include speech and articulation problems requiring speech therapy. There may be a benefit of Prompts for Restructuring Oral Muscular Phonetic Targets, which is an approach that utilizes touch cues to the jaw, tongue, and lips to manually guide word formation, especially in thouse individuals with apraxia of speech.

Management by a lymphedema therapist may be helpful in due to accumulation of fluid, especially in the feet of obese individuals with PWS. This also may require special hygiene and placement of compression stockings. Lymphedema is the most common side effect of growth hormone in adults.

## 14 CARE COORDINATION

An important member of the treatment team is the care coordinator, often nurses, or social workers, whose responsibilities include: coordinating a care plan, assisting in coordinating appointments with the different specialists, improving communication between healthcare providers, ensuring that patients can get to their appointments, educating patients and their families or caregivers, and assisting if there are problems with insurance. They can also assist in finding community resources and support communication with schools and employers.

Patients that come to the clinic locally are seen every 4–6 months depending on age due to growth hormone monitoring and often behavioral management. On a consultation basis when care is coordinated with home‐based specialists, patients are most commonly seen on a yearly basis.

## 15 TELEMEDICINE

To leverage the rapid pace of information technology advancement, several institutions have implemented multidisciplinary telemedicine initiatives. These have generally met with much success, at a minimum saving money, which includes travel burden, offering access to specialized care in rural areas, and improving care for several disorders. For example, by implementing telemedicine, multidisciplinary lung cancer teams were more easily able to consult with thoracic surgeons, reducing mean time between a clinic visit and surgery by five days and saving three work weeks over a one‐year period (Davison et al., 2004). Even greater impacts have been seen when patients have been able to leverage multidisciplinary expertise from their own homes. Through the use of telemedicine, a multidisciplinary team was able to provide a level of dementia care in an underserved retirement community normally only available in major metropolitan areas (Tso, Farinpour, Chui, & Liu, 2016).

Similarly, through implementation of telemedicine in the developing world, multidisciplinary cleft palate teams delivered speech therapy to their patients postsurgery, improving speech in nearly every category (Glazer et al., 2011). Improved technology in this area could be used to administer home‐based therapies such as applied behavioral therapy or to conduct outcome measures in a comfortable environment for the participant.

The difficulties lie in the lack of reimbursement for these services, lack of infrastructure to provide these services, difficulty performing detailed clinical assessments that are improving as the field advances, and challenges of medical licensure to provide multi‐state services (LeRouge & Garfield, 2013).

## 16 HOME‐BASED HEALTH SERVICES

Home‐based and mobile health services are invaluable. They reduce the financial burden on healthcare systems and allow for assessment of the home settings and real‐time incorporation of key environmental controls (Gomes, Calanzani, Curiale, McCrone, & Higginson, 2013; Song, Hill, Bennet, Vavasis, & Oriol, 2013). Therapists and specialists may assess the milieu to recommend modification for ease of activities of daily living and exercise. This is essential for individuals in rural areas suffering complications of PWS. For example, difficulties in traveling have been noted in individuals with morbid obesity and lymphedema, as well as severe anxiety and stubbornness. Assessments in the home may be more accurate with respect to behaviors, which may be masked when entering a clinic for care. With the goal of reaching all families and ensuring quality care is achieved in all underserved areas, home‐based health services and mobile clinics, in which the specialists come to the families or families come to a local centralized location, respectively, is a role of the Center of Excellence. Quality of care for such assessments should be a factor more valued in reimbursement practices (Nuckols, Escarce, & Asch, 2013).

## 17 CLINICAL MANAGEMENT

### 17.1 Diet

Diet previously focused on restriction of calories utilizing the Red Yellow Green System for weight management (Evidence category B) (Bonfig, Dokoupil, & Schmidt, 2009; Schmidt, Pozza, Bonfig, Schwarz, & Dokoupil, 2008). However, recent evidence suggests that a higher fat/lower carbohydrate diet may be more beneficial (Miller, Lynn, Shuster, & Driscoll, 2013). However, calorie restriction should still be considered as patients with PWS have decreased energy expenditure (Bekx, Carrel, Shriver, Li, & Allen, 2003). Carbohydrates should be nourishing carbohydrates such as vegetable and grains. We typically recommend avoiding sugars and artificial sweeteners, drinking water, providing protein at every meal, and incorporating the appropriate amount of fiber based on age (average 20 g). Our patient experience suggests these management changes are modifying the natural history of the disease.

It is difficult to persuade individuals with PWS to consume water. We often recommend infusing flavors from fresh vegetables or fruits such as cucumbers to provide flavor.

Anecdotal reports from families suggest that there are benefits to the ketogenic diet (Evidence category C). Risks include effects on growth and bone mineralization particularly in children with PWS, who are at increased risk of complications such as osteoporosis, hypoglycemia, and meal calculi (Bakker, Kuppens, et al., 2015; Bergqvist, Schall, Stallings, & Zemel, 2008). Data are unclear whether this is a lifelong commitment to maintain therapeutic benefits.

### 17.2 Supplements

There are no randomized, placebo‐controlled clinical trials of supplements in PWS. All evidence for possible use of these supplements is anecdotal and decisions to try these supplements should be made on an individual basis with special considerations (Table 1). It seems prudent to perform double‐blind placebo‐controlled trials to assess the benefits of off label use of these supplements. Additional important methods of tracking success with supplements and medications are through a common data model such as the Global PWS Registry. Evidence‐based guidelines for the use of supplements in individuals with PWS are needed to standardize therapy and determine if benefits exist.

#### 17.2.1 Coenzyme Q10

Coenzyme Q10 (CoQ10) is a key component of the mitochondrial respiratory chain and is required for the generation of adenosine triphosphate (ATP). It also serves as an antioxidant. Studies indicate that low plasma levels of CoQ10 present in individuals with PWS are comparable to levels found in obesity in general, and the use of CoQ10 may be beneficial (Butler et al., 2003; Eiholzer et al., 2008). Its use may improve the duration of suckling in infants and improve stamina in up to 20% of patients (Evidence category C). The stratification of responders and nonresponders remains a gap in medical knowledge. The dose used in the literature is 100‐200 mg/day. No placebo‐controlled large studies are available to assess the benefit of supplementation.

#### 17.2.2 Carnitine

Carnitine is essential for shuttling of long‐chain fatty acids across the mitochondrial membrane for β‐oxidation. Manifestations of carnitine deficiencies include liver, skeletal, and cardiac manifestations and hypoketotic hypoglycemia. Few children with PWS have low serum levels; however, muscle biopsy studies are not suggestive of a deficiency (Butler et al., 2003). A trial of l‐carnitine has not been shown in randomized trials to have benefit. It may help development, but a more critical appraisal is needed (Ma et al., 2012). Carnitine deficiency may be linked to nonsyndromic autism (Beaudet, 2017). The recommended dose is 50 mg kg^‐1^ day^‐1^ divided into two doses (Evidence category C).

#### 17.2.3 Medium‐chain triglyceride oil

Medium‐chain triglycerides (MCTs) provide the liver with an energy source, thereby reducing fat deposition as adipose tissue by bypassing chylomicron formation to lymphatic transport. A diet high in MCT may affect the transformation of inactive to active (acetylated) ghrelin (Kawai et al., 2017). In PWS, ghrelin is elevated throughout the nutritional phases, and more recent studies suggest a role for the active form in disease pathogenesis (Beauloye et al., 2016; Kweh et al., 2015). Studies in overweight individuals indicate that MCT oil supplementation may reduce appetite and food intake (St‐Onge et al., 2014). Anecdotally, MCT oil supplementation may also decrease appetite in children with PWS (Evidence Category C). Formal clinical trials are needed. We start at 0.5 ml every other feeding, advance to every feeding, and increase in increments of 0.25–0.5 ml/feeding at intervals of 2–3 days as tolerated to a goal of one teaspoon dosed throughout the day. MCT has calories and therefore should be incorporated into the individuals diet to ensure a balanced diet.

#### 17.2.4 Multivitamin

Individuals on restricted diets are at risk of deficiencies in essential nutrients (Lindmark, Trygg, Giltvedt, & Kolset, 2010). Gummy vitamins should be avoided due to overall deficient minerals in these options, risk of abuse, and calorie content of gummies. In infants, poly‐vi‐sol® is often used. Other preferable options include multivitamin powders or unpalatable chewable vitamins.

B12 (oral or injections) is indicated for low serum levels, macrocytic anemia, and individuals following a vegan diet.

Docosahexaenoic acid (DHA) is indicated for individuals on a low‐fat diet or those with hyperlipidemia. DHA or essential fatty acid deficiency can arise in individuals on a vegetarian restricted diet. Fish oil has been proposed to decrease risk of some psychiatric disorders, although evidence is conflicting (McGorry et al., 2017; Pawelczyk, Grancow‐Grabka, Kotlicka‐Antczak, Trafalska, & Pawelczyk, 2016).

#### 17.2.5 *N*‐acetylcysteine (Evidence category C)

A small, open‐label study of *N*‐acetylcysteine (NAC) supplementation indicated that this may be effective at curtailing skin picking (Miller & Angulo, 2014). Placebo‐controlled trials are needed. When purchasing, individually wrapped medications should be used to avoid decreased efficacy of the medication over time. Caution should be exercised as there may be side effects while receiving NAC with other psychiatric medications.

### 17.3 Medication considerations

Use of the following medications requires a physician's clinical judgment and decisions should be made individually based on careful consideration of known risks, benefits, and alternatives in light of current best evidence (Table 2). An important note is the hypersentivity to medications noted in many individuals with PWS requiring caution in dosing especially for psychopharmacology. Anesthesia, for example, requires special measures prior to and during anesthesia.

**Table 2:** Evidence to support medication/supplement management in PWS

Medical intervention	Category	Indication	Special Considerations	References
Growth hormone	A	All patients throughout life for short stature, maintenance of lean body mass, cognitive benefits	Potential risk of worsening OSA and link to sudden death (Tauber, Diene, Molinas, & Hebert, 2008) Recommend polysomnography pre–post initiation	Bakker et al. (2017), Bakker, Kuppens, et al. (2015), Bohm, Ritzen, and Lindgren (2015), Coupaye et al. (2016), Dykens, Roof, Hunt‐Hawkins (2017), Hoybye (2015), Kuppens et al. (2017), Lo et al. (2015), Longhi et al. (2015)
HCG	C	Infant males with undescended testes/possibly in puberty	Transient increase in testosterone	Bakker, Wolffenbuttel, et al. (2015), Eiholzer et al. (2007)
Testosterone	C	Male hypogonadism	Potential increased aggression; consider initiating low disease and increasing slowly over time	Kido et al. (2013)
Estrogen/progesterone	C	Female hypogonadism	Potential worsening behavior and increased risk of blood clots; consider initiating low doses and increasing slowly over time	Eldar‐Geva et al. (2013)
Modafinil	B	Excessive daytime sleepiness/narcolepsy and impulsive behavior	Increased risk of severe skin rash can worsen anxiety in some, but some parents report improvement	De Cock et al. (2011), Weselake et al. (2014)
Topiramate	C	Severe skin picking	In neurodevelopmentally disabled children, associated with cognitive slowing	Shapira et al. (2004), Shapira et al. (2002), Smathers et al. (2003)
SSRIs	C	stubbornness, cognitive rigidity, anxiety, and OCD	Threshold of affect, start low and increase slowly	Dech and Budow (1991), Kohn, Weizman, and Apter (2001), Selikowitz, Sunman, Pendergast, and Wright (1990)
Antipsychotics	B	Aggression, impulsivity, magical thinking, psychosis	Increased risk of weight gain with some	Akca and Yilmaz (2016), Araki et al. (2010), Bonnot et al. (2016), Durst, Rubin‐Jabotinsky, Raskin, Katz, & Zislin (2000a, 2000b ), Dykens and Shah (2003), Elliott et al. (2015)
Metformin	B	Insulin resistance	GI side effects, lactic acidosis	Chan, Feher, and Bridges (1998), Miller, Linville, and Dykens (2014)
GLP‐1R agonists	B	Insulin resistance, obesity	Generally not recommended for age <14, GI side effects and possible pancreatitis, slows gastric emptying	Fintini et al. (2014), Salehi et al. (2016)
*N*‐acetylcysteine	C	Skin picking	Individuals seem to develop a tolerance	Miller and Angulo (2014)
Carnitine	C	Carnitine deficiency; Vegetarian/vegan diet	GI distress can be a side effect	Ma et al. (2012), Miller, Lynn, Shuster, and Driscoll (2011)
CoQ10	C	Poor suck, low stamina	GI side effects	Butler et al. (2003), Eiholzer et al. (2008), Miller et al. (2011)
MCT oil	C	Infants with failure to thrive despite adequate calories, weight control in combination with exercise	Additional calories, clogs NG tubes	Ma et al. (2012)
DHA	C	Low‐fat diet, hyperlipidemia	GI side effects	No evidence
B12	C	Low serum vitamin B12 levels, elevated mean corpuscular volume (MCV) and low energy levels, vegetarian/vegan diet	Can increase anxiety	No evidence
Bariatric surgery	D	Life‐threatening obesity, resistant to all other interventions	Increased risk of severe surgical morbidity and mortality	Fong, Wong, Lam, and Ng (2012), Marceau and Biron (2012), Scheimann, Miller, and Glaze (2017)

Note

Medications are listed with the indication and as applicable evidence‐based references. Categories of evidence—A: Good evidence to support a recommendation for use; B: Moderate evidence to support a recommendation for use; C: Poor evidence to support a recommendation for or against use; D: Moderate or good evidence to support a recommendation against use; and E: Good evidence to support a recommendation against use.

CoQ10: coenzyme Q10; DHA: docosahexaenoic acid; GH: growth hormone; GLP‐1R: glucagon‐like peptide‐1 receptor; HCG: human chorionic gonadotropin; MCT: medium‐chain triglycerides; OCD: obsessive–compulsive disorder; PWS: Prader–Willi syndrome; SSRI: selective serotonin reuptake inhibitor.

#### 17.3.1 Growth hormone (Evidence category A)

Growth hormone is standard of care in childhood and should be started at the time of diagnosis, preferably prior to age 1. It was approved by the US Food and Drug Administration in June 2000 for the treatment of growth failure. It does not curtail hyperphagia; however, it promotes improved quality of life, including greater strength and decreased body mass index (BMI) in conjunction with a controlled diet (Hoybye, Hilding, Jacobsson, & Thoren, 2003). It should be titrated based on the IGF1 and IGFBP3 levels. Emerging literature supports use in adults for bone health and promotion of lean muscle mass (Mogul et al., 2008). Early treatment with rGH improves growth and body composition resulting in better motor strength and function. Additional benefits include more favorable lipid profiles, positive effects on development and cognition, and improved depressive symptoms (Bakker, Kuppens, et al., 2015; Dykens, Roof, & Hunt‐Hawkins, 2017; Lo et al., 2015). Contraindications to use include severe obesity, untreated severe obstructive sleep apnea (OSA), uncontrolled diabetes, active malignancy, and active psychosis.

#### 17.3.2 Human chorionic gonadotropin (Evidence category C)

Human chorionic gonadotropin stimulates production of gonadal steroid hormones by stimulating the Leydig cells to produce androgens. It may help undescended testes descend. In a study of 16 boys with PWS, HCG helped testes descent in 23% of cases, 62% reached a lower position, while 76% still required surgical intervention (Bakker, Wolffenbuttel, Looijenga, & Hokken‐Koelega, 2015). It is started around 3 months old. If testes do not descend by 18 months, surgical intervention is recommended. It may also have the added benefit of transiently increasing testosterone levels and promoting penile growth in infant males with micropenis. In adolescents, there may be a benefit of virilization and increased lean muscle mass (Eiholzer, Grieser, Schlumpf, & l'Allemand, 2007).

#### 17.3.3 Hormone replacement therapy (Evidence category C)

Hormone replacement therapy (HRT) with testosterone and estrogen/progesterone replacement therapy is used to augment development of secondary sex characteristics and improve bone mineral density for those with hypogonadism. One caveat is the potential impact on behavior, particularly in boys receiving testosterone. In a small study of boys with PWS with a baseline low score on the Modified Overt Aggression Scale, therapy reduced the percent body fat and increased bone mineral density and lean body mass (Kido et al., 2013).

Additional medication considerations in girls include case‐by‐case consideration of treatment with estrogen, cyclic progesterone, intrauterine devices, or contraceptive pills. Inhibin B and anti‐Mullerian hormone are potential markers of fertility in girls as well as sertoli cell function in boys (Eldar‐Geva et al., 2013). Treatment with either testosterone or estrogen should be started at low doses and increased slowly over time to mimic pubertal development as well as to minimize potential adverse effects.

#### 17.3.4 Modafinil (Evidence category B)

Modafinil is a stimulant medication indicated in the treatment of narcolepsy and is thought to work as a weak dopamine reuptake inhibitor that requires interaction with the dopamine transporter. It plays a role in activation of phasic dopamine signaling. It is often started at the time of presentation of attention and behavioral concerns. Modafinil improved sleepiness based on the Epworth sleepiness scale (De Cock et al., 2011). The recommend starting dose is 100 mg. Rare but serious side effects of this medication are severe dermatologic adverse reactions including Stevens‐Johnson syndrome. Pitolisant is a new medication that has been approved in Europe for the treatment of narcolepsy. In a randomized controlled trial to compare these treatments in narcolepsy, there was no benefit of pitolisant compared to modafinil (Dauvilliers et al., 2013). A randomized controlled trial is needed to assess the benefit of pitolisant in individuals with PWS.

For treatment of attention deficit, impulsivity and hyperactivity trials of the nonstimulants atomoxetine (Stratterra) and alpha‐agonists guanfacine and clonidine may be an appropriate first step. Other considerations include stimulant medications such as amphetamine or methylphenidate preparations. It is also important to consider evaluation and treatment of OSA in consideration of behavioral concerns.

#### 17.3.5 Topiramate (Evidence category B)

Topiramate is reported to help with skin picking behaviors (Shapira, Lessig, Lewis, Goodman, & Driscoll, 2004; Shapira, Lessig, Murphy, Driscoll, & Goodman, 2002; Smathers, Wilson, & Nigro, 2003). However, this medication can cause significant cognitive dulling, which is problematic in patients with intellectual disabilities (Mula, 2012).

#### 17.3.6 Selective serotonin reuptake inhibitors (Evidence category C)

Often psychiatrists recommend a trial of selective serotonin reuptake inhibitors (SSRI) medication to ameliorate stubbornness, cognitive rigidity, anxiety, and obsessive–compulsive behaviors. A trial of SSRIs such as citalopram may be beneficial. Although clinical data show that in low doses these medications may be helpful, our experience suggests there may be a threshold after which they exacerbate symptoms. Slow titration upward to the desired effect is recommended with a safe, tapered discontinuation if the medication is not beneficial. Pharmacogenetic testing is now available to help clinicians make more informed choices as to which psychiatric medication may be best for each individual patient. However, the validity of these tests is still limited.

#### 17.3.7 Antipsychotics (Evidence category C)

There are no randomized controlled trials to guide psychiatric medications. Consideration of these medications should be based on the physicians’ clinical judgment and on an individuals’ symptoms such as aggression and psychosis. A more comprehensive discussion of psychiatric medications can be found in separate reviews (Bonnot et al., 2016; Dykens & Shah, 2003). Use of a global registry with documentation of medication use, dosing in PWS, and positive and negative effects to bridge this clear gap in knowledge is essential toward appropriate psychopharmacology.

## 18 CLINICAL TRIALS

### 18.1 Lessons learned

Nusinersen has transformed the natural history of spinal muscular atrophy (Chiriboga et al., 2016). It has unequivocally modified the motor development in all types of SMA. The success of this targeted therapy with a clear molecular rationale provides an important lesson regarding clinical trials. Ideally, one would know the molecular basis for the therapeutic target, have clear outcome measures, and Centers of Excellence would collaborate to work with industry to run double‐blind placebo‐controlled randomized clinical trials.

The search for a treatment that unambiguously modifies the natural history of PWS and ameliorates distressing characteristics such as hyperphagia and skin picking continues. While some trials have shown promise, others have illuminated the safety concerns of clinical trials. For example, beloranib is a selective methionine aminopeptidase 2 inhibitor. In a phase 3, randomized, placebo‐controlled clinical trials, patients with PWS had a −9.45% placebo‐adjusted change in body weight. In addition, there was a clinically significant decrease in hyperphagia (McCandless et al., 2017). Unfortunately, two patients with PWS enrolled in the treatment group experienced fatal pulmonary emboli. Further analysis of all clinical data showed an increased risk of thromboembolic events in all patients receiving this medication. This drug has been discontinued, but provides a noteworthy lesson in the importance of transparent communication with participating drug companies and potentially considering the pathogenic rationale for the use of the drug in PWS.

Rimonabant is another example of a potentially promising but failed medication for reduction of hyperphagia. Rimonabant was shown to be successful for the treatment of obesity, presumably by causing decreased appetite and lipogenesis, and increased energy expenditure. In rats, it prevents ghrelin release in fasting and postprandial states and inhibits the release of growth hormone (Alen et al., 2013). However, a clinical trial of this medication in PWS was terminated early due to adverse psychiatric effects in adults with PWS (Motaghedi et al., 2011).

The challenges of doing clinical trials in PWS are offset by the high unmet medical need and burden of disease of a disorder that affects about 1/15,000–1/30,000 people. Additionally, medications targeted at individuals with PWS may apply to the treatment of obesity in a much broader populace. However, it must be considered that individuals with PWS have unique characteristics including a high pain tolerance, decreased muscle mass, increased predisposition to psychiatric disorders, delayed gastric emptying, decreased tolerance for physical activity, and slow metabolism which may cause unforeseen complications. These experiences highlight the need for clear measures of the benefit‐to‐risk ratio. Validated PWS‐specific outcome measures and biomarkers are key to assessing objective success of a drug to avoid drug side effects associated with medications likely causing a placebo effect. The multidisciplinary clinic and Center of Excellence play an important role in facilitating randomized clinical trials and in providing an ongoing evaluation of individuals enrolled in clinical trials and continued consideration of the risk versus benefit of interventions in a population with a very high unmet medical need for effective therapies.

### 18.2 Ongoing and upcoming clinical trials

#### 18.2.1 Oxytocin/Carbetocin

Individuals with PWS have fewer oxytocin neurons in the paraventricular nucleus of the hypothalamus, and studies have shown elevated oxytocin plasma and cerebrospinal fluids levels (Johnson, Manzardo, Miller, Driscoll, & Butler, 2016; Martin et al., 1998; Swaab, Purba, & Hofman, 1995). It is hypothesized that disrupted oxytocin signaling and feedback plays a role in the symptoms of hyperphagia, anxiety, autistic‐like features, and obsessive–compulsive behaviors. A double‐blind randomized placebo‐controlled trial showed improved trust in others, and less tendency to sadness 45 min after treatment. Behavior improved in the 48 hr following treatment and data also suggested less conflict with others in the 12 hr post‐treatment (Tauber et al., 2011). Eighty‐eight percent of infants assessed by the Neonatal Oral‐Motor Scale normalized sucking behavior after treatment with intranasal oxytocin (Tauber, Diene, & Molinas, 2016). This correlated to videofluoroscopy, acetylated (active) ghrelin, and improved connections in the right superior orbitofrontal network (Mogul et al., 2008). Additionally, improvements in the Clinical Global Impression scale scores and mother–infant interactions were observed and a decrease in social withdrawal behavior. Studies show contradictory data regarding the impact of oxytocin on temper outbursts with dose increases (Einfeld et al., 2014). Trials are in phase II, and initial observations suggest that oxytocin is effective particularly in limiting anxiety and compulsive behaviors (Miller et al., 2017). A phase II trial of carbetocin, which acts more specifically as an agonist at peripheral oxytocin receptors, has also been completed. A phase III trial of carbetocin is close to recruitment.

#### 18.2.2 Diazoxide choline

Diazoxide has been used to treat hyperinsulinism in infants and children. A long‐acting formulation (diazoxide choline) is under investigation to treat hyperphagia and aggressive behaviors. The medication's possible ability to improve sensitivity to leptin and insulin, particularly in Agouti‐related protein (AgRP) and pro‐opiomelanocortin (POMC) neurons in the hypothalamus, prompted clinical trials in PWS. A phase I study showed decreased hyperphagia, reduced fat mass and weight, and improved resting energy expenditure. It also improved antisocial behaviors (per press release). A phase III study is underway.

#### 18.2.3 Cannabidiol oral solution (CBD oil)

Anecdotally, families report a positive affect particularly on behavior. A Phase II trial is in process. A recent study suggests caution should be exercised when purchasing products purported to contain CBD oil as only 30% of products were accurately labeled including about 43% that were underlabeled and about 26% that were overlabeled. Tetrahydrocannabinol was detected in about 20% of samples (Bonn‐Miller et al., 2017).

#### 18.2.4 Glucagon‐like peptide 1 receptor agonists

Glucagon‐like peptide 1 (GLP‐1) receptor agonists (GLP1RA) such as exenatide and liraglutide were initially introduced for treatment of type II diabetes. GLP1RAs promote weight loss and curb appetite, and are candidates for treating hyperphagia and obesity. GLP1Rs reduce gastric emptying and have central effects on appetite. However, reduced gastric emptying is of concern in PWS, and an increased risk of gastric rupture in an already at‐risk population with increased pain tolerance should not be ignored. GLP1RAs decrease circulating ghrelin (Gagnon, Baggio, Drucker, & Brubaker, 2015). Use in PWS patients showed decreased appetite scores (Salehi et al., 2017), antidiabetogenic effects, and improved body mass index (BMI), particularly in the first 12 months of therapy, and the medication was safe when used in adults with PWS. In addition, it improved satiety and decreased circulating ghrelin in one patient (Fintini et al., 2014).

#### 18.2.5 Melanocortin‐4 receptor agonist

Heterozygous and homozygous mutations in melanocortin‐4 receptor (MC4R) cause a human obesity syndrome. The MC4R receptor agonist RM‐493 increased resting energy expenditure (REE) in obese adults (Chen et al., 2015). Early trials in obese patients with heterozygous mutations in MC4R and other mutations in the hypothalamic leptinmelanocortin signaling pathway, as well as in individuals with PWS, seem promising. A phase II trial of this medication in PWS has been completed. Data showed no benefit of Setmelanotide in PWS (Miller et al presented at PWSA meeting).

#### 18.2.6 Unacylated ghrelin analog (AZP‐531)

Unacylated ghrelin analogs inhibit plasma acylated ghrelin levels and improve glucose metabolism. Results from a Phase II trial are promising and a randomized, double‐blind placebo‐controlled study showed decreased appetite, food‐seeking behavior, and smaller waist circumference (Allas & Abribat, 2013). A trial to look at ghrelin‐O‐acyltransferase (GOAT) inhibition, which will block the acetylation of ghrelin, is underway.

## 19 DISCUSSION

A multidisciplinary care model with expertise in genetics, endocrinology, nutrition, pulmonology and sleep, behavior, and interventional services with care coordination is helpful to ensure an integrated and evidence‐based approach to care of individuals with PWS. The center of excellence is a resource to the patients, families, pharma, and the medical community. It serves to accelerate translational research to improve characterization of the phenotypic features of PWS, and advance understanding of the molecular pathogenesis toward developing future targeted therapies. The gaps in knowledge and lack of evidence‐based treatments exemplify the importance of these centers to perform translational research, establish objective biomarkers and outcome measures, and perform randomized placebo‐controlled trials. A functioning common data model agreed upon by all stakeholders including the parents, medical providers, and pharma would accelerate research and advance knowledge in the field.

## CONFLICT OF INTEREST

Dr. Duis consults with Disruptive Nutrition and is a sub‐investigator on clinical trials funded by GLWL and Soleno Pharmaceuticals. Dr. Scheimann completed research funded by Zafgen Pharmaceuticals. Current sources of funding include NIMH and Foundation for Prader‐Willi Research. Dr. Salehi has completed research funded by Zafgen Pharmaceuticals. She is currently working with Soleno Pharmaceuticals. Current sources of funding to Dr. Miller include the Prader‐Willi Syndrome Association USA and the Foundation for Prader‐Willi Research. Dr. Shoemaker has completed research funded by Zafgen Pharmaceuticals and served on their hypothalamic obesity advisory board. She is participating in clinical trials funded by Soleno and GLWL Pharmaceuticals. Current sources of funding to Dr. Shoemaker include AstraZeneca, Novo Nordisk, Rhythm Pharmaceuticals, and NIDDK K23 DK101689. Dr. Miller completed research funded by Zafgen Pharmaceuticals, Ferring Pharmaceuticals, and Rhythm Pharmaceuticals. She is participating in clinical trials funded by Soleno and GLWL Pharmaceuticals. Drs. van Wattum, Bingham and Ms. Brokamp, Ms. Childers, and Ms. Fairbrother report no financial or potential conflict of interests.
